# Do beluga whales truly migrate? Testing a key trait of the classical migration syndrome

**DOI:** 10.1186/s40462-023-00416-y

**Published:** 2023-08-30

**Authors:** Luke Storrie, Lisa L. Loseto, Emma L. Sutherland, Shannon A. MacPhee, Greg O’Corry-Crowe, Nigel E. Hussey

**Affiliations:** 1 Centre for Earth Observation Science, Department of Environment and Geography, The University of Manitoba, Winnipeg, MB Canada; 2 Freshwater Institute, Fisheries and Oceans Canada, Winnipeg, MB Canada; 3 Harbor Branch Oceanographic Institute, Florida Atlantic University, Fort Pierce, FL USA; 4 Department of Integrative Biology, University of Windsor, Windsor, ON Canada

**Keywords:** Migration, Nomadism, Telemetry, Cetaceans, Beluga whale, Foraging

## Abstract

**Background:**

Migration enables organisms to access resources in separate regions that have predictable but asynchronous spatiotemporal variability in habitat quality. The classical migration syndrome is defined by key traits including directionally persistent long-distance movements during which maintenance activities are suppressed. But recently, seasonal round-trip movements have frequently been considered to constitute migration irrespective of the traits required to meet this movement type, conflating common outcomes with common traits required for a mechanistic understanding of long-distance movements. We aimed to test whether a cetacean ceases foraging during so-called migratory movements, conforming to a trait that defines classical migration.

**Methods:**

We used location and dive data collected by satellite tags deployed on beluga whales (*Delphinapterus leucas*) from the Eastern Beaufort Sea population, which undertake long-distance directed movements between summer and winter areas. To identify phases of directionally persistent travel, behavioural states (area-restricted search, ARS; or Transit) were decoded using a hidden-Markov model, based on step length and turning angle. Established dive profiles were then used as a proxy for foraging, to test the hypothesis that belugas cease foraging during these long-distance transiting movements, i.e., they suppress maintenance activities.

**Results:**

Belugas principally made directed horizontal movements when moving between summer and winter residency areas, remaining in a Transit state for an average of 75.4% (range = 58.5–87.2%) of the time. All individuals, however, exhibited persistent foraging during Transit movements (75.8% of hours decoded as the Transit state had ≥ 1 foraging dive). These data indicate that belugas actively search for and/or respond to resources during these long-distance movements that are typically called a migration.

**Conclusions:**

The long-distance movements of belugas do not conform to the traits defining the classical migration syndrome, but instead have characteristics of both migratory and nomadic behaviour, which may prove adaptive in the face of unpredictable environmental change. Such patterns are likely present in other cetaceans that have been labeled as migratory. Examination of not only horizontal movement state, but also the vertical behaviour of aquatic animals during directed movements is essential for identifying whether a species exhibits traits of the classical migration syndrome or another long-distance movement strategy, enabling improved ecological inference.

**Supplementary Information:**

The online version contains supplementary material available at 10.1186/s40462-023-00416-y.

## Background

Long-distance movement strategies have evolved in response to spatiotemporal fluctuations in environmental conditions and variation in the selective pressures exerted on an animal throughout ontogeny [1–3]. Predictable and asynchronous spatiotemporal variability in habitat quality between two or more regions favour migration [2]. Moving between two or more consistent and distinct home ranges, often over a seasonal cycle, can be highly adaptive if it enables greater exploitation of resources at successive sites of predictable quality than at intermediate sites or remaining at one site year-round [3–5]. Unpredictable spatiotemporal variability in resources that are patchily distributed over large areas instead favours nomadism [6], whereby individuals benefit from searching for and responding to resources, resulting in unpredictable trajectories among individuals or among years for an individual [3].

Diverse taxa across the animal kingdom have been shown to exhibit a consistent suite of traits associated with migratory movements, termed the migratory syndrome [5]. Based on the definition of Kennedy ([7], p. 8): “Migratory behavior is persistent and straightened-out movement effected by the animal’s own locomotory exertions or by its active embarkation upon a vehicle. It depends on some temporary inhibition of station keeping responses, but promotes their eventual disinhibition and recurrence”. We refer to this definition as “classical migration”. Therefore, a key behavioural trait of the migration syndrome is that during an animal’s persistent and straightened out-movements, maintenance activities required for growth and reproduction are suppressed, and consequently a truly migrating animal is undistracted by local resources such as food and mates that would normally evoke a response [5, 7]. Migrants may undertake brief ‘stopovers’ at predictable sites between summer and winter residency areas primarily for recovery or energy accumulation on route to their destination [8–10], but during directed movements in the migratory phase the animal is undistracted and ceases feeding [2, 5]. Numerous experimental and observational studies on terrestrial species such as insects and birds have found strong support for the migratory syndrome (see [5, 7, 11] for examples). Over the past two decades, however, seasonal round-trip movements between discreet residency areas have often been considered to constitute migration irrespective of the suite of traits required to meet the classical definition of this movement type [12–14]. Established in seminal literature [2, 7, 15], focusing on the common behavioural and physiological traits underlying long-distance movements, rather than their ecological outcomes is key to providing a mechanistic understanding of the causes and consequences of movement strategies [16–18], i.e., what makes an individual migratory versus non-migratory [7]. Furthermore, understanding whether a species undertakes classical migration (i.e., displays multiple traits of the migration syndrome) or exhibits characteristics of other long-distance movement strategies such as nomadism is critical as these strategies differ in their success depending on spatiotemporal predictability of resources [3, 4], their stability under environmental change, and how measures should best be applied for conservation management [19–21].

Due to difficulties in observing animals during their long-distance movements, the behavioural traits of the classical migration syndrome have been difficult to test for many species until the proliferation of animal telemetry [22, 23]. Animal telemetry, electronic tags deployed on animals that transmit data to receivers (typically satellites or acoustic receivers), has enabled analysis of movement trajectories in two-dimensional space and has revealed irregular horizontal movements in species traditionally considered as migratory ([6] and references therein). But directionally persistent horizontal movement tracks between seasonal residency areas, classified through various state-space models applied to movement data, are frequently referred to as migrations (e.g., [24–27]). Marine animals move in a three-dimensional environment, yet in labelling movement phases as migrations, studies rarely examine vertical movements that are essential for testing whether an animal conforms to the traits of the migration syndrome, i.e., straightened out and undistracted movements with maintenance activities suppressed. This is especially notable in endothermic marine megafauna, for which recent studies have shown that deep, vertical excursions, including foraging behaviour, can occur during directionally persistent horizontal movement tracks [28, 29].

The infraorder Cetacea includes species that exhibit some of the longest distance movements in the animal kingdom, often referred to as migrations, as well as species that are considered nomads and residents [30–32]. Many populations exhibit directionally persistent and seasonal low to high latitude long-distance movements, indicative of migrations, linked to spatiotemporal variation in foraging opportunities and pressures related to predation and temperature requirements of calves [1, 33, 34]. But recent studies have revealed complexities among cetacean movements. Some species exhibit partial migrations depending on age, sex, and reproductive status [30, 35]. Aseasonal movements provide evidence of the importance of moulting as a major driver behind long-distance movements [27]. Non-migratory populations or portions of populations exist within species that otherwise are considered to migrate [32, 36, 37]. Cetaceans can use predictable stopover sites [10, 38], but evidence of exploratory behaviour and supplemental feeding during what are traditionally considered as migratory movements [39–41] raises questions about whether these movements conform to the migration syndrome (i.e., classical migration).

The current study aims to test the assumption that perceived migratory movements in a highly mobile cetacean are undistracted and maintenance activities are suppressed, following the traits defining classical migration. By examining movements in three-dimensions using location and dive data collected by long-term satellite tags deployed on beluga whales (*Delphinapterus leucas*) from the wide-ranging Eastern Beaufort Sea (EBS) population, which undertake > 2000 km long distance movements between summer and winter areas [42, 43], we tested the hypothesis that beluga whales cease feeding during so-called migratory movements using established dive profiles as a proxy for foraging. Our intention is to reiterate the importance of the mechanisms that drive classical migration, rather than purely defining the outcome of a movement type.

## Materials and methods

### Data collection

**Fig. 1:**
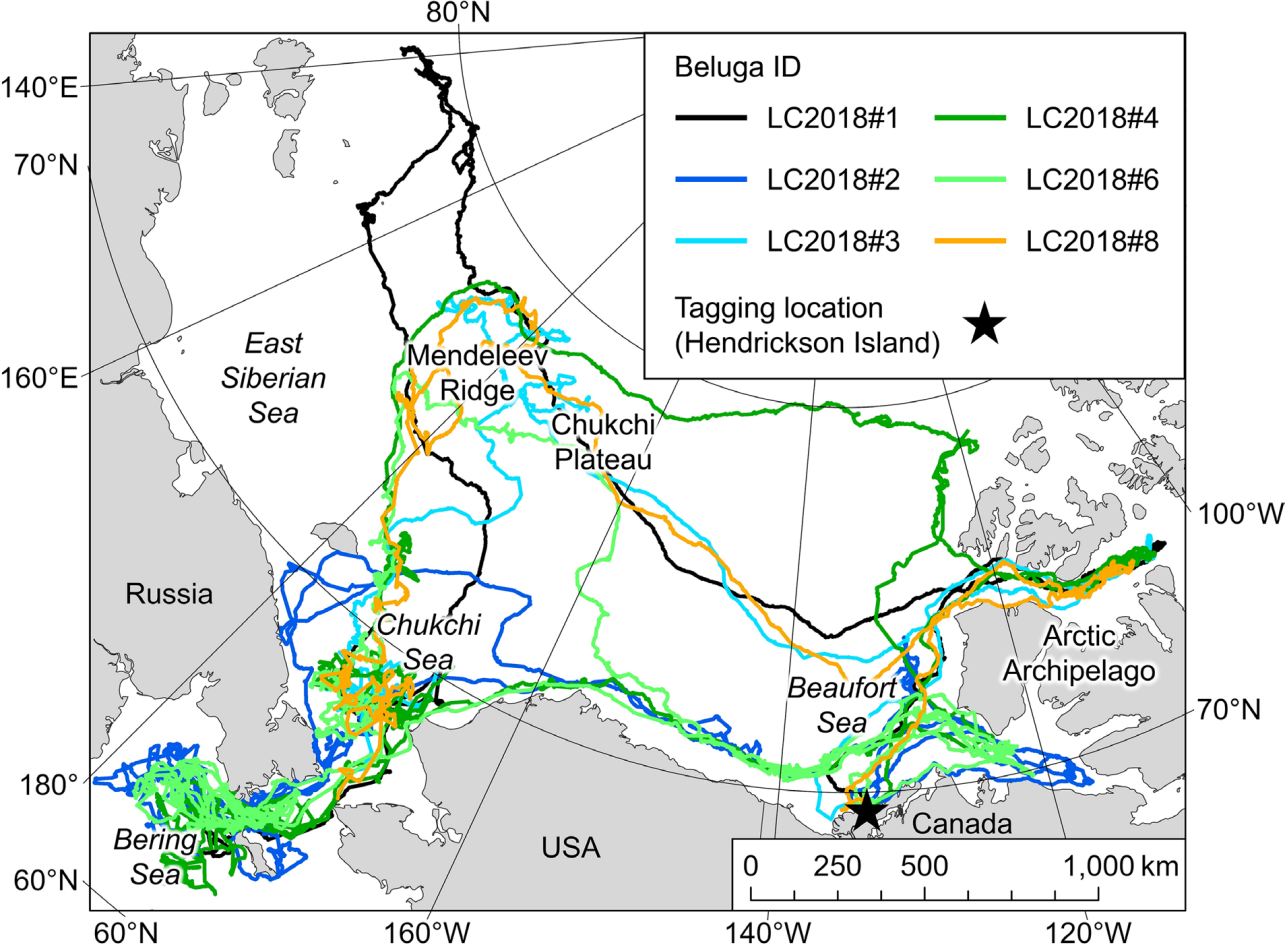
Map of the study area showing full tracks for the six belugas

Belugas from the EBS population were tagged at Hendrickson Island, Northwest Territories, Canada (Fig. 1) in July 2018 and 2019, as part of a project co-developed with the Inuvialuit; details on tagging methods can be found in Storrie et al. [44]. For this study we used data from six male belugas tagged in 2018 equipped with SPLASH10-F-238 tags (Wildlife Computers Ltd., Redmond, WA, United States), as these belugas transmitted data for 161–357 days covering long-distance movements between seasonal residency areas in fall (n = 6) and spring (n = 3). Tags sampled depth (± 0.5 m) at 1 s intervals and transmitted the data as hour-long time series messages of depths subsampled at 75 s intervals. Tags also collected Fastloc-GPS locations (hereafter GPS), and Argos locations were estimated each time a transmission was made. A range of tag programming setups were used to inform on the relationship between the settings and tag longevity for future studies (see [44] for details of tag programming). In brief, tags were programmed with higher transmission limits and to collect GPS locations (at 7–30 min intervals) every day from July through September (400–1040 transmissions per day) and switched to have lower transmission limits with GPS locations collected every 7th day from October/November onwards (175–740 transmissions per day). Three of the belugas were double tagged with a MiniPAT (pop-up archival tag) (Wildlife Computers Ltd., Redmond, WA, United States), which archived and then transmitted depth time series data after release from the animal. MiniPAT data were used to fill in missing depth data from the SPLASH10-F-238 tags (Table 1).

### Location processing

Strict filtering of the location data and continuous-time correlated random walk (CTCRW) models [45] were used to estimate locations over hourly intervals with the greatest accuracy possible. GPS locations calculated using < 5 satellites and/or with a residual value > 35 were removed [46], and remaining GPS locations were assigned semi-major- and semi-minor axis errors of 50 m and an ellipse orientation of 0°. Argos locations that had a semi-major axis error > 3 km were removed. Argos locations were then filtered by speed (5 m/s) and turning angle (15° and 25°, unless spikes were < 2500 and 5000 m, respectively) [47] using the fit_ssm function in the *foieGras* package v0.7-6 [48] in R v 4.1.2 [49]. Further Argos locations were removed if they occurred within 10 min of a GPS location. Argos and GPS locations were then combined, and locations were removed if they had a semi-major axis error > 500 m and occurred within 20 min of a location with a semi-major axis error of ≤ 500 m. Tracks were then split into segments when there were gaps > 6 h, and track segments were removed if they contained < 4 locations and/or covered < 3 h. After filtering the location data, remaining locations within each segment occurred 43.8 min apart on average. CTCRW models were fit to each track segment using the fit_ssm function [48] and locations predicted at hourly intervals. Diagnostic plots were checked and all CTCRW-modeled tracks were viewed to confirm movement trajectories had been modeled appropriately (i.e., did not contain a modeled location that would require unrealistic speeds or turning angles between high accuracy locations in the raw filtered data).

### Modelling movement state

A hidden Markov model was fit using the fit_hmm function in the *momentuHMM* v1.5.4 [50, 51] package in R, to decode behavioural states at each hourly CTCRW-modeled location. Step length (modeled with a gamma distribution) and turning angle (modeled with a Von Mises distribution) were used in a two-state model; area-restricted search (ARS) (slow and tortuous movements) or Transit (fast and directed movements). A two-state model was selected to allow for simple biological interpretation between movement states typically associated with foraging, resting, or social behaviour (ARS) vs. migrating or commuting behaviour (Transit) [24–26, 39, 52]. Each track segment was assigned a unique ID and models were fit using all track segments for all individuals. A histogram of step lengths was plotted to inform on initial parameters in the first model, *m1*, following recommendations by Michelot et al. [53]. These were set at means of 1 and 5 km, and standard deviations of 2 and 3 km for ARS and Transit, respectively. Initial parameters for turning angle in *m1* were set at a concentration of 1 for ARS (i.e., high frequency of turning angles) and 13 for Transit (persistence in direction). A second model, *m2*, was fit using the parameter estimates from *m1* as the initial parameters. To check whether different initial parameters resulted in convergence at different parameter estimates, two additional models, *m3* and *m4*, were fit with initial parameters for step length and turning angle that were less and greater than the parameter estimates from *m1*, respectively. A further model, *m5*, was fit with extreme values for the step length initial parameters, using the minimum step length as the mean for the ARS state and the maximum step length as the mean for the Transit state. Full details on parameters can be found in Supplementary Material 1. The model with extreme initial parameters, *m5*, converged at biologically meaningless parameter estimates and transition probabilities (ARS mean step length = 0.006 km/hr, transition probability from Transit to ARS = 0.000), so was rejected. All other models converged at identical parameter estimates and transition probabilities, so *m1* was selected (Supplementary Material 1). The stateProbs function in *momentuHMM* was used to calculate the probability of each location being in either of the states; state-decoded locations with a probability of < 0.9 of being ARS or Transit were labeled as Uncertain and excluded from the analyses.

### Distinguishing long-distance movements vs. seasonal residency phases

EBS belugas undertake some of the longest distance movements among this species, that are often referred to as migrations [44, 54, 55]. Overwintering at lower latitudes in the Bering Sea, EBS belugas move northeastwards in spring to form summer congregations in estuaries and feed in deep offshore areas, before travelling west in fall [42–44]. To test our hypothesis related to migration, we first needed to isolate the arena in which belugas undertake long-distance movements by removing known seasonal residency regions where shorter directed movements between local resource patches can occur. This was achieved by creating 50% utilization distrbution (UD) polygons from the locations which had been state-decoded as ARS, separately for the six months centered on summer (April-September, bandwidth = 75 km) and winter (October-March, bandwidth = 100 km), using the *adehabitatHR* v0.4.19 package [56] in R (see details in Supplementary Material 2). Polygons were extended to the nearest land barrier to prevent short-term movements by belugas outside of the 50% UDs but within establshed seasonal residency areas [42, 43] being defined as occurring during the long-distance movement phase. State-decoded locations which occurred within these polygons were excluded from analyses and all remaining locations are hereafter referred to as occurring during the long-distance movement phase.

### Testing whether maintenance activities are suppressed during long-distance movements

EBS beluga dives were previously characterized by time and depth metrics, classified into types, and likely functions identified for each type [44]. Four dive types had time-depth structures that indicated they were used principally for foraging: Deep Benthic, Deep Pelagic V, Deep Pelagic W, and Intermediate Benthic. These dives are hereafter referred to as foraging dives. The designation of foraging dives was based on the depths of these dives (median max. depth for these dive types ranged from 50.5 to 576.0 m), as well as other components of their time-depth structure such as descent and ascent rates (see [44] for full details). Alternative functions for deep dives can relate to species’ responses to predators at shallow depths, whereby they may socialize [57] or sleep [58] at greater depths. Killer whales (*Orcinus orca*), the only fully aquatic predator of belugas, are increasingly moving north of the Bering Strait [59], but given their relative scarcity and the high frequency of deep dives by belugas (e.g., 16,531/90,211 dives analysed in [44] were to depths > 101 m), we considered that predator avoidance is unlikely the primary function of these four dive types. These four foraging dives are also unlikely to represent an optimal transiting depth to avoid surface wave drag, the impacts of which apply at depths less than three times an animal’s body diameter [60] (equivalent to a depth of ~ 3 m in belugas). Deep skewed-shaped dives, where an animal uses its buoyancy to passively drift through the water column, may be used by marine mammals in sleeping, resting or facilitating digestion [61, 62]. The one skew-shaped dive that was identified in EBS belugas [44] was proposed to have foraging as its most likely function, but this was excluded from the current analyses due to uncertainty around whether drifting through the water column could represent another function. The total number of foraging dives was calculated for each hour and assigned to each state-decoded location based on the time stamp. Hours were removed if they did not contain complete dive data, i.e., if an hour of depth time series data was followed by an hour of missing data and a dive crossed the two hours it could not be characterized, so the depth time series data from that dive was removed and the hour in which it started classified as incomplete (see [44] for details). To test whether belugas cease foraging during long-distance movements typically labeled as migratory, we calculated the total number of foraging dives that started during each hourly location that was state-decoded as Transit, and compared this to the number of dives that occurred during locations that were state-decoded as ARS for reference. This was calculated separately for each of the two periods of long-distance movements; Fall and Spring.

## Results

### Long-distance movements

Tagged belugas initiated their westward movements between mid-August and early September but there was variability in the routes taken (Fig. 1) and distances travelled (2498–6030 km, Table 1). Three belugas took similar routes over the deep waters of the Arctic Basin towards the Chukchi Plateau (LC2018#1, LC2018#3 and LC2018#8). One beluga went north before heading west (LC2018#4), and two belugas headed west via a more southern route along the Beaufort Slope (LC2018#2 and LC2018#6). Two belugas spent several weeks around the Chukchi Plateau and Mendeleev Ridge (LC2018#3 and LC2018#8), whereas one beluga passed through this region and went 700 km further west (LC2018#1). All belugas entered the winter residency area in the Chukchi Sea between mid-October and mid-November. The three belugas that transmitted data into their Spring long-distance movements took more direct routes towards the summer residency area in April and May, travelling distances of 832–1079 km (Table 1), past the northwest coast of Alaska and along the Beaufort Slope.

**Table 1:** Information on deployments and number of state-decoded hourly locations (number of state-decoded locations with complete dive data in parentheses) available for each beluga during the long-distance movement phase. Long-distance movement phase track length (km) is based on the (path) distance between consecutive CTCRW estimated locations for the fall and spring long-distance movements (i.e., sequence of locations outside the summer and winter residency areas) calculated using the geosphere v1.5-14 package [63] in R. This is a minimum estimate of travel distance as calculations are based on the shortest distance between consecutive points according to the ‘Vincenity (ellipsoid)’ method, and gaps ≤ 12.75 days were present between individual CTCRW track segments

Beluga ID	PTT	Beluga length (m)	Tag deployment date range	Duration (days)			State-decoded hourly locations during the long-distance movement phase (number of hours with complete dive data in parentheses)				Long-distance movement phase track length (km)	
							Total	ARS	Transit	Prop. Transit (%)	Fall	Spring
**LC2018#1**	174965	4.20	03/07/18–02/01/19	184			1252 (345)	283 (87)	969 (258)	77.4	6030	NA
**LC2018#2**	174967	4.70	04/07/18–19/06/19	351			319 (61)	65 (21)	254 (40)	79.6	2498	832
**LC2018#3**	174962	4.06	06/07/18–15/12/18	163			1136 (466)	145 (60)	991 (406)	87.2	5649	NA
**LC2018#4**	174963	4.44	08/07/18–07/06/19	335			1363 (435)	456 (182)	907 (253)	66.5	4986	1079
**LC2018#6**	174966	4.40	08/07/18–29/06/19	357			773 (120)	321 (66)	452 (54)	58.5	3329	917
**LC2018#8**	174969	4.25	09/07/18–19/12/18	164			995 (166)	169 (34)	826 (132)	83.0	5444	NA
**Total**					1554		5838 (1593)	1439 (450)	4399 (1143)			

**Fig. 2:**
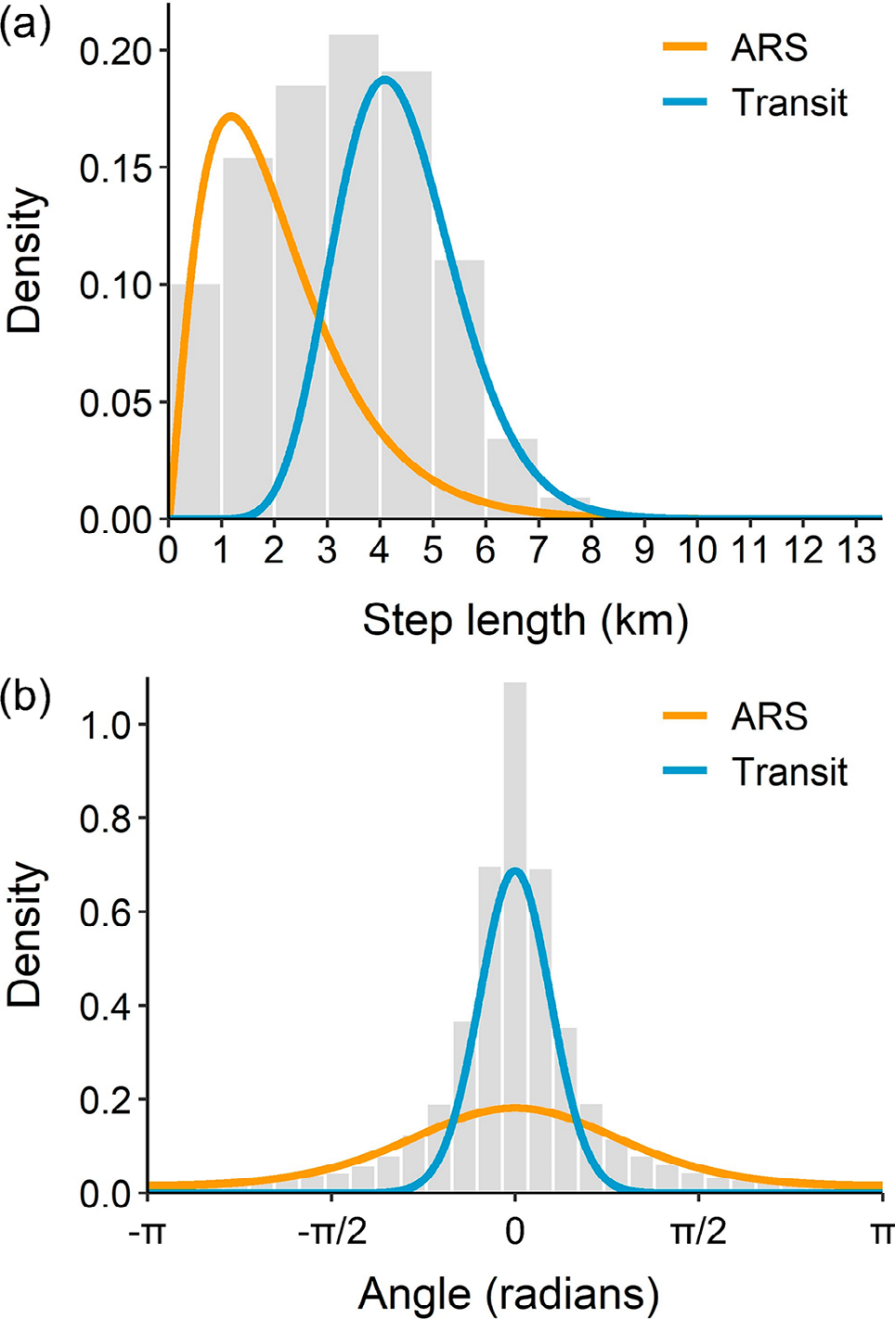
Estimated probability distributions for (a) step length and (b) turning angle, for the two-state (ARS and Transit) HMM. Grey bars show histograms of the input data for each of these parameters

### Horizontal movement behavioural states

A total of 20,014 locations estimated at hourly intervals from the CTCRW models were available for decoding states using the HMM. Parameter estimates indicated locations decoded as the ARS state were characterized by slow speeds (mean step = 2.09 km, sd = 1.37 km, Fig. 2a) and frequent directional changes (angle concentration = 1.22, Fig. 2b), whereas Transit locations had higher speeds (mean step = 4.39 km, sd = 1.15 km, Fig. 2a) and fewer directional changes (angle concentration = 10.97, Fig. 2b). After removal of state-decoded locations with a probability < 0.9 of being in either state, 16,620 locations remained. A total of 5,373 of these locations contained complete dive data, and of these, 1,593 were recorded during the long-distance movement phase (1,224 in Fall, and 369 in Spring), enabling an assessment of whether foraging dives occurred during perceived migratory movements (Table 1). Belugas recorded an average of 75.4% (range = 58.5–87.2%) of the time in a Transit state during the long-distance movement phase (Table 1), compared with 39.1% (range = 27.6–45.5%) during summer and winter residency phases.

### Foraging behaviour during perceived migratory movements

**Fig. 3:**
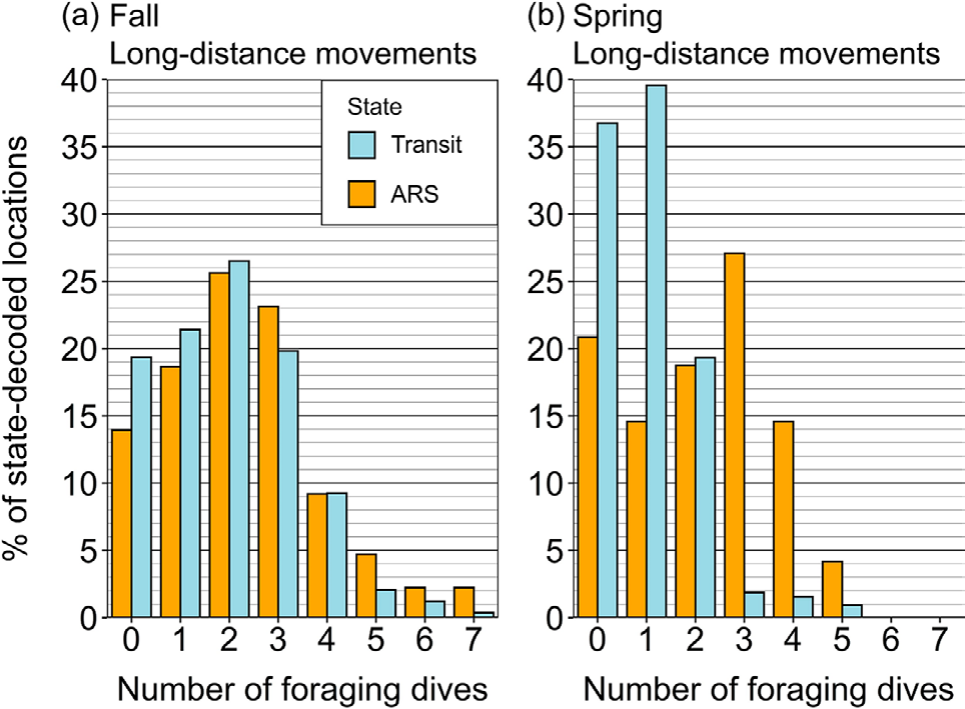
Percentage of hours where a given number of foraging dives [44] were recorded for the locations decoded as Transit or ARS while belugas were in the long-distance movement phase in (**a**) Fall and (**b**) Spring

While belugas were in the long-distance movement phase, foraging dives were recorded during the majority of hours decoded as Transit in both Fall and Spring (80.7% and 63.3% of hours with ≥ 1 dive, respectively, Fig. 3). Foraging dives were frequent while belugas were in the Transit state during Fall (e.g., 59.2% of hours with ≥ 2 dives) and comparable to the number of dives during ARS movements (67.4% of hours with ≥ 2 dives), whereas during spring belugas tended to make fewer dives during Transit than ARS movements (e.g., 23.7 vs. 64.6% of hours with ≥ 2 dives).

**Fig. 4:**
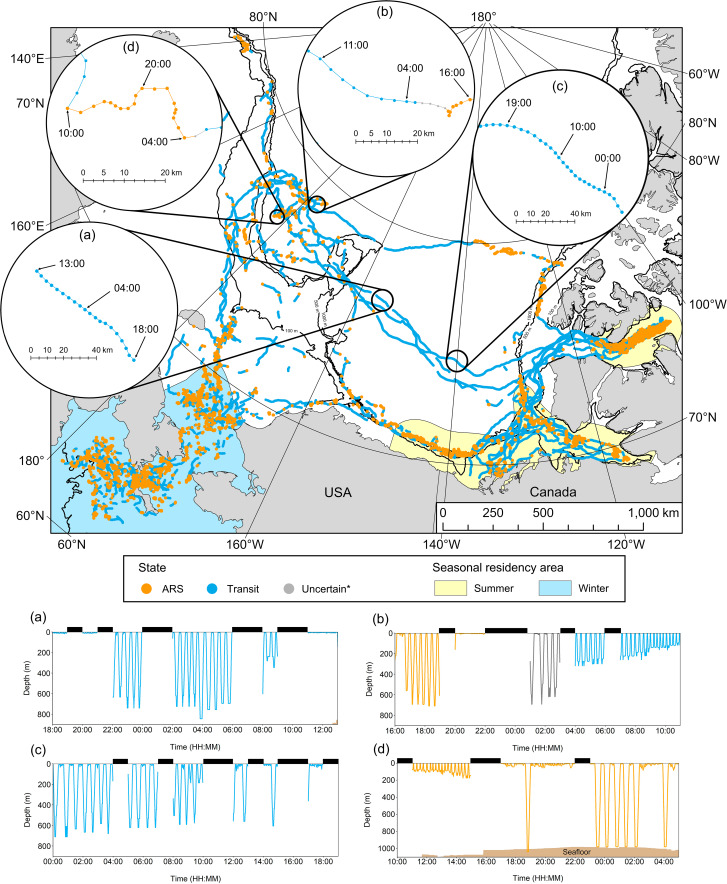
State-decoded locations recorded by six belugas between July 2018 and June 2019. The long-distance movement phase analysed includes all locations not bounded by the summer or winter residency area polygons. ‘Uncertain*’ locations (probability of < 0.9 of being in either the ARS or Transit state), when present, shown in zoomed inset panels only. Zoomed inset panels **a**-**d** show examples of state-decoded beluga tracks, with the corresponding dive profiles colour-coded by state shown in the lower panels. (**a**) beluga LC2018#3, 1st -2nd Sep 2018, (**b**) beluga LC2018#3, 13th -14th Sep 2018, (**c**) beluga LC2018#1, 6th Sep 2018 (**d**) beluga LC2018#8, 6th Sep 2018. Black bars above dive profiles denote periods with missing depth data. All times are given in UTC. Zoomed inset panels only show the track of the individual beluga referred to for that period. Seafloor depth is denoted by brown shading when within the range of depths shown on the y-axis

Representative examples of belugas making foraging dives whilst in a Transit state during fall can be seen in Fig. 4, identifying this population actively searches for and/or responds to resources during long-distance movements between summer and winter residency areas. From 1st September at 18:00 to 2nd September at 13:00 (19 h), beluga LC2018#3 was in a Transit state over a distance of 78 km and recorded 18 dives to between 240 and 840 m (Fig. 4a), with 3 h of data where no dives were made. From 13th September at 16:00 to 22:00 (6 h) beluga LC2018#3 was in an ARS state over a distance of 10 km; it recorded 9 dives to between 160 and 710 m, but also remained at depths ≤ 12.5 m over a two-hour period (Fig. 4b). It then entered an Uncertain state for four hours, recording 5 dives to > 290 m, before entering a Transit state over 40 km between 03:00 and 12:00 (9 h) on 14th September, undertaking 24 dives to depths > 100 m (Fig. 4b). Beluga LC2018#1 was in a transit state on 6th September (Fig. 4c); between 00:00 and 19:00 (19 h) it traveled 75 km and recorded 23 dives to between 170 and 710 m. Foraging dive frequency was comparable during transit movements and ARS movements (Fig. 4d). The above examples each had 4–8 h of dive data missing during which additional foraging dives may have been made. Foraging dives were made during the Transit state whilst belugas were in the long-distance movement phase during both fall and spring; further examples of state-decoded tracks and corresponding dive profiles can be found in Supplementary Material 3.

## Discussion

Migration is defined as a movement syndrome with distinct characteristics (i.e., predictable, directional and undistracted movements [5, 7]) that is shared among diverse species [64]. Here we demonstrate that a highly mobile marine predator, beluga whales from the EBS population, undertake predominantly directionally persistent horizontal movements (i.e., Transit state) between discrete summer and winter residency areas, typically associated with migratory behaviour [24, 26, 65]. But whilst doing so, belugas persistently exhibit deep vertical excursions characteristic of foraging and/or exploratory behaviour; more akin to a nomadic strategy whereby animals search for and respond to resources during long-distance movements [6]. These data highlight that long-distance movements and an assigned transient state derived only from horizontal movement data should not be assumed to represent classical migration, where movements are undistracted and maintenance activities are suppressed [5, 7, 11]. Classifying these movements as migration ultimately underestimates the behavioural ecology of a given species. It would appear that the long-distance movements of beluga whales include traits of both migration and nomadism, but conform wholly to the classical views of neither [2–4, 6], which may be more ubiquitous across aquatic predators than previously thought.

Migration and nomadism both allow species to exploit spatially heterogeneous resources, but whilst seasonal predictability in the spatial distribution of resources favors migration, spatiotemporal unpredictability of resources favors nomadism [3, 6]. Consequently, these two movement strategies require different behavioural and physiological traits to access resources [5]. Migrants require memory mechanisms (through genetics, experience, or communication) to ensure that they arrive at and/or depart from a site to coincide with known resource abundance or deterioration [3, 66]. Nomads use exploratory behaviour and respond to local conditions to locate patchily distributed and unpredictable resources [3, 6]. Migrants also rely more on prior deposition of energy reserves, primarily as lipids, and morphological adaptations to minimize the cost of movement than nomads due to the greater distances travelled and the longer non-feeding periods that must be endured [5].

Persistent foraging/exploratory behaviour by EBS belugas during their long-distance movements coupled with differences in routes taken among individuals could suggest that belugas adopt a nomadic strategy after departing a seasonal residency area, searching for and responding to resources while moving in the general direction of their next seasonal residency area. Irregularly fluctuating resources over large geographic areas lead to unpredictable and spatially variable nomadic movement patterns among individuals and within an individual across years [3, 4]. Reconciling components of both migratory and nomadic strategies over a seasonal cycle within an individual requires separate predictable and unpredictable features over the range of EBS belugas. In certain situations, sea ice can be a predictable feature, constraining beluga distribution south of the Bering Strait in winter [42] while land-fast sea ice prevents movements into estuaries and the Arctic Archipelago until summer [67–69]. In contrast, interannual variability in sea ice concentration and the position of the ice edge throughout the Arctic Basin and Beaufort Slope [70, 71], where belugas undertake their long-distance movements during fall and spring, are less predictable. These regions, however, can be highly productive if somewhat ephemeral, dependent on sea ice concentration, upwelling storms, and daily light cycles, and may support abundant foraging opportunities by cetaceans [72, 73]. Given a temporal mismatch between optimal conditions in summer and winter residency areas; the flexibility in movement strategies among beluga populations (residents [74, 75] vs. migrants [43, 76]); and their ability to detect prey over large distances [77], EBS belugas may benefit from switching to a nomadic strategy (exploring and responding to resources, [6]) in this unpredictable intermediate region rather than moving directly between summer and winter areas.

Recent studies have acknowledged a continuum of movement strategies between the traditional resident/nomadic/migratory trichotomy [4, 6, 78, 79], and foraging behaviour during directional movements that have traditionally been labeled as migrations is not uncommon. Notably, several bird and bat species exhibit a ‘fly-and-forage’ strategy, where individuals frequently switch between feeding and flying during long-distance movements towards the next residency area [80, 81]. This represents an alternative strategy to classical migration, either opportunistically to minimize prolonged stopovers [80], or an adaptation to reduce the amount of energy that must be deposited prior to migration [82]. EBS belugas have a thick blubber layer (10–15 cm) prior to their fall migration and a thinner layer (~ 5 cm) following their spring migration [83], indicating some deposition of energy reserves prior to long-distance movements, but their persistent foraging behaviour while transiting has similarities to the ‘fly-and-forage’ strategy. This suggests that the continuum of long-distance movement strategies found in birds (fly-and-forage, stopovers, non-stop classical migration, [14]) may also be present in cetaceans.

Foraging behaviour during traditionally assumed migratory movements between core residency areas may be more common in cetaceans than is currently acknowledged. For instance, whilst female and immature sperm whales (*Physeter macrocephalus*) remain at subtropical latitudes year-round and exhibit nomadic behaviour [84], males undertake long-distance seasonal movements to higher latitudes [30, 85]. But partially digested prey in the stomachs of males caught during spring and fall [86] suggest they feed during these movements. Equally, killer whales (*Orcinus orca*) tagged in the Southern Ocean make aseasonal long-distance movements to lower latitudes likely to facilitate moulting [27], during which they recorded dives (mean depth = 37.5 m, individual maxima = 182–380 m) at depths unlikely to represent optimal transiting [60]. Given the findings of the present study, we suggest a closer examination of dive behaviour during cetaceans’ traditionally inferred migratory movements is required to identify whether foraging has been underestimated at these times.

Foraging during directed movements would seem less likely for the mysticetes, which are typically larger than odontocetes and so have lower costs of transport [87, 88], can undertake long fasting periods [89], but rely on thresholds of prey density for energetically efficient foraging [90, 91]. Evans & Bearhop [14] recently drew from terminology used to organize reproductive strategies to propose a framework organizing migratory strategies along a continuum from capital migration (no feeding during long-distance movements, conforming to classical migration) to income migration (regular feeding during long-distance movements). The cetaceans generally fit well into this framework; the mysticetes are typically capital breeders (use stored energy during reproduction) and the odontocetes are income breeders (feed during reproduction) [92]. Given that these reproductive strategies depend on an animal’s ability to store energy and that equivalent size mysticetes and odontocetes have vastly different seasonal energy reserve requirements [89], it is unsurprising that many of the mysticetes are considered migratory [32], whereas the odontocetes include more species which are year-round resident, nomadic, or partially migratory (e.g., [84, 85, 93, 94]). But the traditional view of mysticetes undertaking a “feast or famine” approach over an annual cycle (i.e., classical migration); foraging intensively at high latitudes during summer to build up energy reserves to fuel their long-distance movements and reproduction during other times of the year, has recently been brought into question by evidence of a continuum of movement strategies within this group [32]. For instance, humpback whales (*Megaptera novaeangliae*) in the southwest Pacific have been observed feeding outside of their summer residency areas [95], and may remain in an ARS state for > 30 days in regions along their migration route [39]. Blue whales (*Balaenoptera musculus*) and fin whales (*Balaenoptera physalus*) tagged in the North Atlantic frequently switched between transiting and ARS movements during their migrations [52, 65]. Differences in the location and frequency of ARS movements among individuals in both species [52, 65], and the use of ARS movements by blue whales progressing northwards [52], are more akin to nomadic and march-and-browse strategies, respectively, than migrations with predictable stopover sites [4, 14, 65]. Furthermore, whilst other studies on mysticetes have shown little or no ARS behaviour along migration routes [96], it is typically assumed that foraging does not occur during a transiting (directed) state, which may not be the case.

Recent studies on the Arctic cetaceans have revealed shifts in long-distance movement strategies associated with changes in sea ice: narwhals and belugas delay their fall migrations as the ice-free season lengthens [55, 97] and bowhead whales have been identified overwintering in their typical summer ground [98]. The success of shifts in the timing of long-distance movements will largely depend on whether the cues for departure from a site are concomitant with suitable environmental conditions at the destination site [99, 100]. EBS belugas tagged in the 1990s and 2000s were reported to make deep dives during their long-distance movements [101], but quantifying foraging during this period was limited by unknown time-depth structures of dives and horizontal movement state (ARS vs. Transit). These historical data, however, suggest foraging behaviour during more recent long-distance movements is unlikely a response associated with changing environmental conditions in the region [102]. We note that the current study only included adult males and EBS belugas segregate by age, sex, and reproductive status [54]. A nearshore long-distance movement route has been observed by Inuvialuit in fall: “when you stand at Shingle Point and look out, you can see whale blows as far as the eye can see, heading toward Alaska. Thousands of them.” (Dennis Arey, Aklavik Hunters and Trappers Committee, pers. comm. to S. MacPhee, October 2022). This route was not taken by belugas in the present study, which went offshore shortly after being tagged, so it is unknown whether the findings of the present study can be applied to the entire population. But, the diversity of movement strategies among beluga populations [43, 74, 75] and the strategy in EBS belugas which includes elements of both migration and nomadism, suggests high plasticity in movement strategies in this species that may provide greater resilience in the face of environmental change when compared with obligate migrants or residents [20, 21]. Strong social bonds and shared movement cultures, as found among belugas [103, 104], are common in nomadic populations [6], whereby individuals in a social group can share information on suitable habitats and foraging success [84]. We posit that with the increasing unpredictability of environmental conditions in the Arctic [105], beluga whales will exhibit more nomadic movements and possibly less predictable summer and winter ranges, which may lead to distinct movement cultures and reproductive isolation among social groups [103, 106].

## Conclusion

Beluga whales from the Eastern Beaufort Sea population exhibit persistent vertical foraging behaviour during horizontal directionally persistent long-distance movements, which contradicts a key trait of the classical migration syndrome, where movements are undistracted and maintenance activities are suppressed. The long-distance movements of belugas have characteristics of both migratory and nomadic behaviour; strategies which rely on opposite ends of a continuum of resource predictability from high to low, respectively. The diversity and flexibility in beluga long-distance movement strategies may prove highly adaptive in the face of unpredictable environmental change.

Long-distance movements in cetaceans are frequently referred to as migrations, but foraging behaviour during these movements may be more common than is currently acknowledged. Labeling the continuum of long-distance movements in cetaceans as migrations potentially conflates common outcomes with common traits. We are not proposing that all long-distance movements in cetaceans include persistent foraging behaviour, but suggest that identifying whether a species exhibits traits of the classical migration syndrome or another long-distance movement strategy such as nomadism is essential for improved ecological inference. This will be critical in the face of increasing unpredictability of the spatiotemporal distribution of resources associated with climate change.

### Electronic supplementary material

Below is the link to the electronic supplementary material.

Supplementary Material 1

Supplementary Material 2

Supplementary Material 3

## Data Availability

The datasets used herein are co-owned by the Department of Fisheries and Oceans Canada and the Inuvialuit (represented by the Game Council, and the Fisheries Joint Management Committee). The collaboration honours the UN Declaration on the Rights of Indigenous Peoples Act and Inuvialuit data sovereignty as they co-lead the program and co-manage this beluga population. The datasets presented in this article are available upon reasonable request. Requests should be directed to LL (Lisa.Loseto@dfo-mpo.gc.ca) and will be reviewed by the Inuvialuit Game Council and Fisheries and Oceans Canada.

## Abbreviations

EBS: Eastern Beaufort Sea
ARS: Area-restricted search
