# Supplementary material for: Do beluga whales truly migrate? Testing a key trait of the classical migration syndrome
Source: Mov Ecol. 2023 Aug 30;11:53. doi: 10.1186/s40462-023-00416-y (PMC10469428; doi:10.1186/s40462-023-00416-y)
Supplement: Supplementary file 1 — Supplementary Material 1 [file 40462_2023_416_MOESM1_ESM.docx]

Supplementary material for the manuscript:

**Do beluga whales truly migrate? Testing a key trait of the classical migration syndrome**

**Authors:** Luke Storrie, Lisa L. Loseto, Emma L. Sutherland, Shannon A. MacPhee, Greg O'Corry-Crowe, Nigel E. Hussey

**Supplementary Material 1:** Hidden Markov model parameters

| Model | Initial parameters | | | | Parameter estimates | | | | Transition probabilities | | | | Initial distribution | |
| --- | --- | --- | --- | --- | --- | --- | --- | --- | --- | --- | --- | --- | --- | --- |
|  | Step mean (sd) | | Angle concentration | | Step mean (sd) | | Angle concentration | | ARS-ARS | ARS-Transit | Transit-ARS | Transit-Transit | ARS | Transit |
|  | ARS | Transit | ARS | Transit | ARS | Transit | ARS | Transit |  |  |  |  |  |  |
| m1 | 1.00 (2.00) | 5.00 (3.00) | 1.00 | 13.00 | 2.09 (1.37) | 4.39 (1.15) | 1.22 | 10.97 | 0.901 | 0.099 | 0.068 | 0.932 | 0.877 | 0.123 |
| m2 | 2.09 (1.37) | 4.39 (1.15) | 1.22 | 10.97 | 2.09 (1.37) | 4.39 (1.15) | 1.22 | 10.97 | 0.901 | 0.099 | 0.068 | 0.932 | 0.877 | 0.123 |
| m3 | 0.9 (3.00) | 2.39 (3.00) | 0.10 | 6.97 | 2.09 (1.37) | 4.39 (1.15) | 1.22 | 10.97 | 0.901 | 0.099 | 0.068 | 0.932 | 0.877 | 0.123 |
| m4 | 4.09 (3.00) | 6.39 (3.00) | 5.22 | 14.97 | 2.09 (1.37) | 4.39 (1.15) | 1.22 | 10.97 | 0.901 | 0.099 | 0.068 | 0.932 | 0.877 | 0.123 |
| m5 | 0.002  (6.00) | 13.22  (6.00) | 1.00 | 13.00 | 0.006  (1.97) | 3.28  (2.00) | 0.57 | 2.37 | 0.009 | 0.991 | 0.000 | 1.000 | 0.000 | 1.000 |

**Table 1:** Candidate HMMs with different initial parameters. m1-m4 all resulted in identical parameter estimates, transition probabilities, and initial distribution, so m1 was selected.
