# Supplementary material for: Do beluga whales truly migrate? Testing a key trait of the classical migration syndrome
Source: Mov Ecol. 2023 Aug 30;11:53. doi: 10.1186/s40462-023-00416-y (PMC10469428; doi:10.1186/s40462-023-00416-y)
Supplement: Supplementary file 2 — Supplementary Material 2 [file 40462_2023_416_MOESM2_ESM.docx]

Supplementary material for the manuscript:

**Do beluga whales truly migrate? Testing a key trait of the classical migration syndrome**

**Authors:** Luke Storrie, Lisa L. Loseto, Emma L. Sutherland, Shannon A. MacPhee, Greg O'Corry-Crowe, Nigel E. Hussey

**Supplementary Material 2:** Methods used to distinguish long-distance movements vs. seasonal residency phases.

1. **Distinguishing migratory vs. residency areas**

EBS belugas occupy regions in the Beaufort Sea and in the west of the Arctic Archipelago during the summer, and the Chukchi and Bering Seas from late fall through winter (1–5) but with variability among individuals (4). To test our hypotheses related to migration, we first needed to broadly remove these known residency areas to discriminate between commuting movements between resources within a core area (6) and long-distance migratory movements. Firstly, the state-decoded locations were separated into summer (April-September) and winter (October-March). We then downsampled the locations which had been state-decoded as ARS (area-restricted search) to one per day (closest to 00:00 UTC) per individual beluga, to remove spatial autocorrelation in relocations. Summer locations were reprojected into UTM zone 9 and winter locations reprojected into UTM zone 2, representative of their spatial distribution. We then used the kernelUD function from the *adehabitatHR* v0.4.19 package (7) in R to plot the 50% utilization distributions separately for the locations for summer and winter, with a grid cell size of 5 km. An initial bandwidth of 50 km was used and increased in 5 km increments until there was no fragmentation within known summer and winter residency areas (8). These were converted into three polygons; one for winter (bandwidth = 100 km), covering the Chukchi Sea and the Bering Sea; and two for summer (bandwidth = 75 km), one covering the Amundsen Gulf and the Beaufort slope, and one covering Viscount Melville Sound, as belugas transit over a distance of ~400 km between these regions, (see Figure 1). To further remove locations which could arise from an animal briefly transiting outside of a core area, we further extended these polygons in the Chukchi Sea (winter polygon extended to the west coast of Alaska and Russia), and the Amundsen Gulf (summer polygon extended to Banks Island and the north coast of Alaska) (Figure 1). State-decoded locations which occurred within these polygons were excluded from analyses, and all remaining locations are hereafter referred to as occurring during the migratory phase.

We also note that the data is biased towards July (n = 6 tags), and data was less complete from January-June (n = 3 tags) but based on previous studies of telemetry and visual examination of the tracks, the areas identified here are the best estimates of summer and winter residency areas.


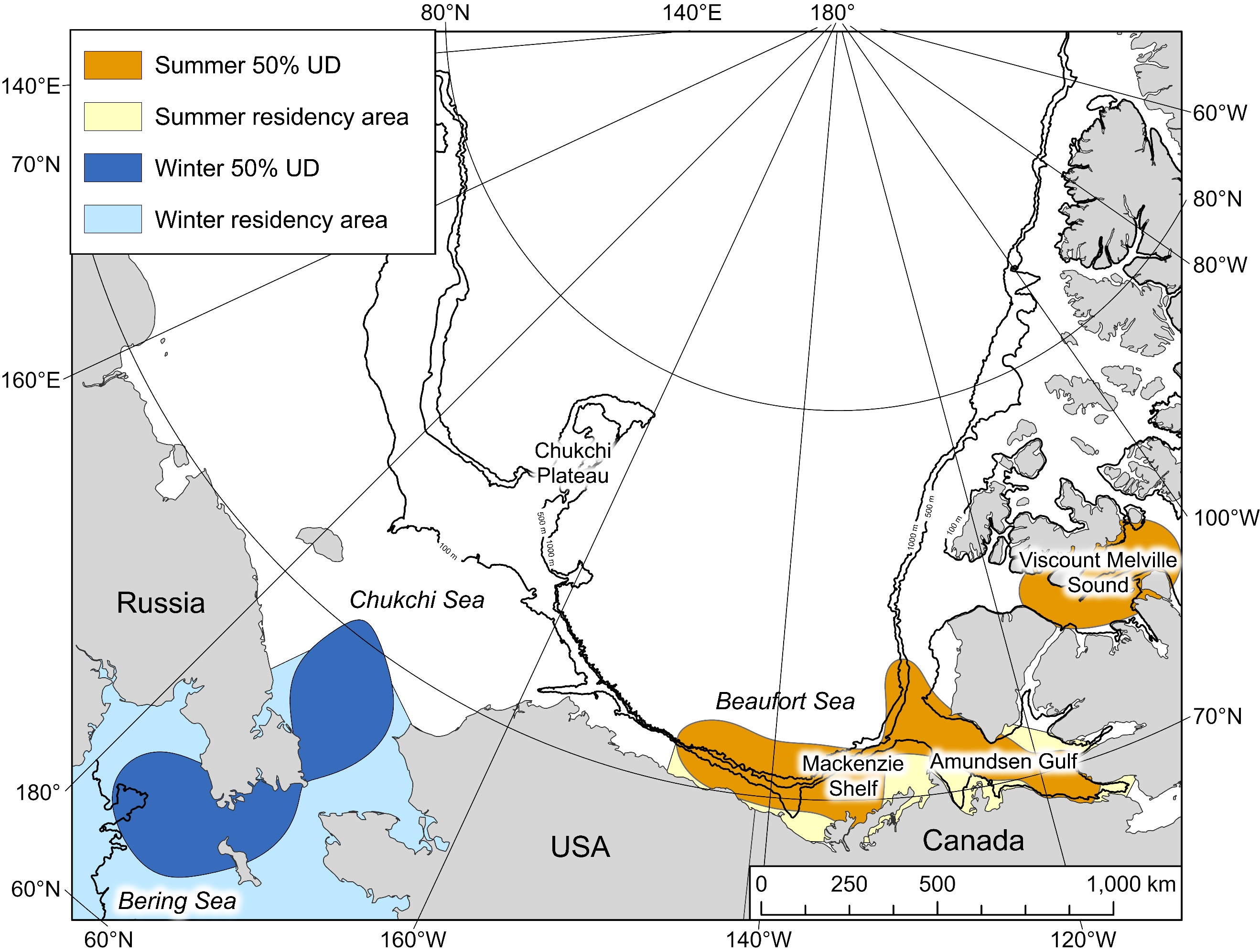


**Figure 1:** Map of the 50 % utilization distributions (UDs) derived from summer and winter locations decoded as the ARS state. Summer and winter residency areas are based on extensions of these polygons to land, to prevent small movements by belugas outside of these polygons being defined as occurring during the migratory phase. The more northerly summer 50% UD polygon was not extended as this already represented a clear boundary.

**References**

1. Storrie L, Hussey NE, MacPhee SA, O’Corry-Crowe G, Iacozza J, Barber DG, et al. Year-Round Dive Characteristics of Male Beluga Whales From the Eastern Beaufort Sea Population Indicate Seasonal Shifts in Foraging Strategies. Front Mar Sci. 2022 Jan 3;8:715412.

2. Citta JJ, Richard P, Lowry LF, O’Corry-Crowe G, Marcoux M, Suydam R, et al. Satellite telemetry reveals population specific winter ranges of beluga whales in the Bering Sea. Mar Mamm Sci. 2017 Jan 1;33(1):236–50.

3. Hauser DDW, Laidre KL, Suydam RS, Richard PR. Population-specific home ranges and migration timing of Pacific Arctic beluga whales (Delphinapterus leucas). Polar Biol. 2014 Aug 1;37(8):1171–83.

4. Loseto LL, Richard P, Stern GA, Orr J, Ferguson SH. Segregation of Beaufort Sea beluga whales during the open-water season. Can J Zool. 2006 Dec;84(12):1743–51.

5. Barber DG, Saczuk E, Richard PR. Examination of Beluga-Habitat Relationships through the Use of Telemetry and a Geographic Information System. Vol. 54, ARCTIC. 2001.

6. Dingle H, Drake A v. What is Migration? Bioscience. 2007;57(2):113–21.

7. Calenge C. adehabitatHR: home range estimation. R package version 0.4.19. [Internet]. 2021 [cited 2022 Jul 8]. Available from: https://cran.r-project.org/web/packages/adehabitatHR/index.html.

8. Kie JG. A rule-based ad hoc method for selecting a bandwidth in kernel home-range analyses. Animal Biotelemetry. 2013 Sep 2;1(1).
