# Supplementary material for: Do beluga whales truly migrate? Testing a key trait of the classical migration syndrome
Source: Mov Ecol. 2023 Aug 30;11:53. doi: 10.1186/s40462-023-00416-y (PMC10469428; doi:10.1186/s40462-023-00416-y)
Supplement: Supplementary file 3 — Supplementary Material 3 [file 40462_2023_416_MOESM3_ESM.docx]

Supplementary material for the manuscript:

**Do beluga whales truly migrate? Testing a key trait of the classical migration syndrome**

**Authors:** Luke Storrie, Lisa L. Loseto, Emma L. Sutherland, Shannon A. MacPhee, Greg O'Corry-Crowe, Nigel E. Hussey

**Supplementary Material 3:** Additional maps showing HMM-decoded states and corresponding dive profiles.

1. **Additional HMM-decoded locations and corresponding dive profiles**


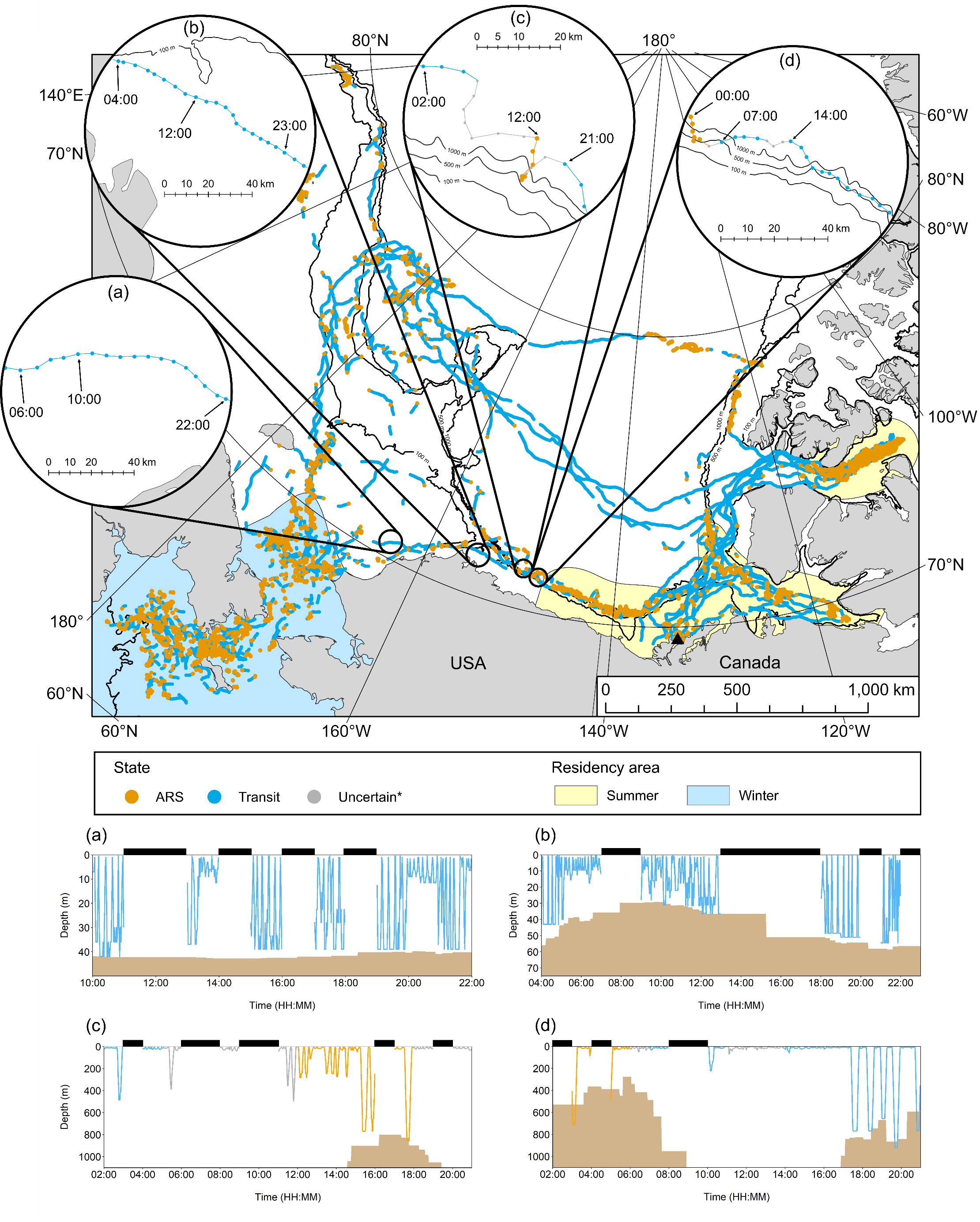


**Figure 1:** State-decoded locations recorded by belugas between July 2018 and June 2019. The long-distance movement phase analysed includes all locations not bounded by the summer or winter residency area polygons. ‘Uncertain*’ locations (probability of < 0.9 of being in ARS or Transit) shown in zoomed inset panels a-d only. Zoomed inset panels a-d show examples of state-decoded beluga tracks, with the corresponding dive profiles colour-coded by state shown in the lower panels. (a) beluga LC2018#2 15^th^ May 2019 at 06:00-22:00 UTC (note, depth data unavailable for several hours prior to 10:00, (b) beluga LC2018#6 29^th^ April 2019 at 04:00 UTC to 30^th^ April 2019 at 01:00 UTC, (c) beluga LC2018#4 1^st^ May 2019 between 02:00-23:00 UTC, (d) beluga LC2018#2 22^nd^ May 2019 at 00:00 UTC to 23^rd^ May 2019 at 01:00 UTC. Black bars above dive profiles denote periods with missing data and the seafloor depth is shaded brown at the bottom. Note, zoomed inset panels only show the individual beluga referred to for that period. Note that the shallowest dives (up to 23 m) were not classified as foraging type dives.


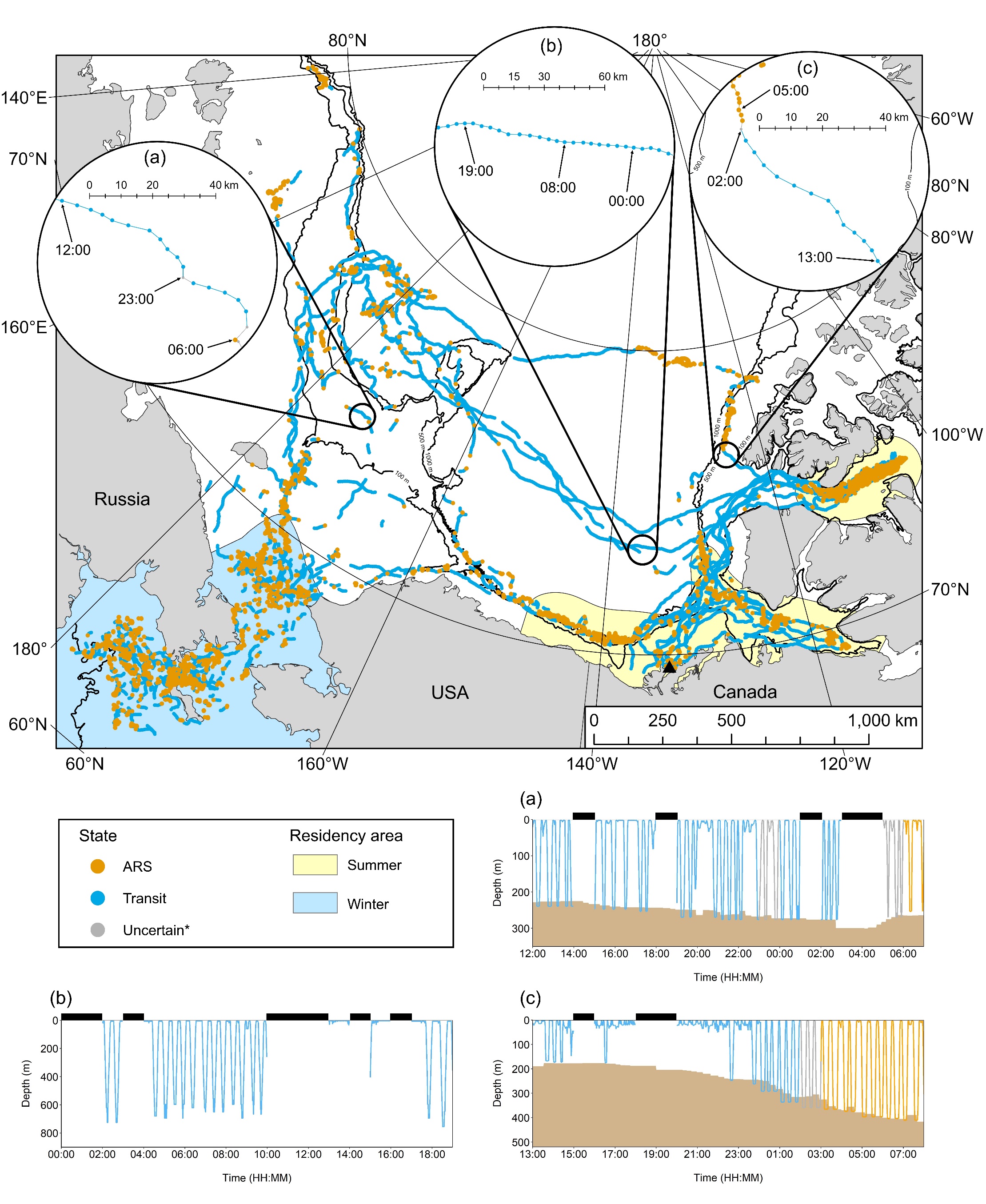


**Figure 2:** State-decoded locations recorded by belugas between July 2018 and June 2019. The long-distance movement phase analysed includes all locations not bounded by the summer or winter residency area polygons. ‘Uncertain*’ locations (probability of < 0.9 of being in ARS or Transit) shown in zoomed inset panels a-c only. Zoomed inset panels a-c show examples of state-decoded beluga tracks, with the corresponding dive profiles colour-coded by state shown in the lower panels. (a) beluga LC2018#1 12^th^ November 2018 at 12:00 UTC to 13^th^ November 2018 at 07:00 UTC, (b) beluga LC2018#3 26^th^ August 2018 00:00-19:00 UTC, (c) beluga LC2018#4 24^th^ August 2018 at 13:00 UTC to 25^th^ August 2018 at 08:00 UTC. Black bars above dive profiles denote periods with missing data and the seafloor depth is shaded brown at the bottom. Note, zoomed inset panels only show the individual beluga referred to for that period. Note that the shallowest dives (up to 23 m) were not classified as foraging type dives.





**Figure 3:** State-decoded locations recorded by belugas between July 2018 and June 2019. The long-distance movement phase analysed includes all locations not bounded by the summer or winter residency area polygons. ‘Uncertain*’ locations (probability of < 0.9 of being in ARS or Transit) shown in zoomed inset panels a-c only. Zoomed inset panels a-c show examples of state-decoded beluga tracks, with the corresponding dive profiles colour-coded by state shown in the lower panels. (a) beluga LC2018#3 15^th^ October 2018 at 06:00 UTC to 16^th^ October 2018 at 01:00 UTC, (b) beluga LC2018#3 10^th^ September 2018 at 09:00 UTC to 11^th^ September 2018 at 04:00 UTC, (c) beluga LC2018#6 12^th^ September at 01:00-19:00 UTC. Black bars above dive profiles denote periods with missing data and the seafloor depth is shaded brown at the bottom. Note, zoomed inset panels only show the individual beluga referred to for that period. Note that the shallowest dives (up to 23 m) were not classified as foraging type dives.





**Figure 4:** State-decoded locations recorded by belugas between July 2018 and June 2019. The long-distance movement phase analysed includes all locations not bounded by the summer or winter residency area polygons. ‘Uncertain*’ locations (probability of < 0.9 of being in ARS or Transit) shown in zoomed inset panels a-c only. Zoomed inset panels a-c show examples of state-decoded beluga tracks, with the corresponding dive profiles colour-coded by state shown in the lower panels. (a) beluga LC2018#3 6^th^ September 2018 at 13:00 UTC to 7^th^ September 2018 at 08:00 UTC, (b) beluga LC2018#1 13^th^ September 2018 at 13:00 UTC to 14^th^ September 2018 at 07:00 UTC, (c) beluga LC2018#4 15^th^ July between 03:00-22:00 UTC. Black bars above dive profiles denote periods with missing data and the seafloor depth is shaded brown at the bottom. Note, zoomed inset panels only show the individual beluga referred to for that period. Note that the shallowest dives (up to 23 m) were not classified as foraging type dives.


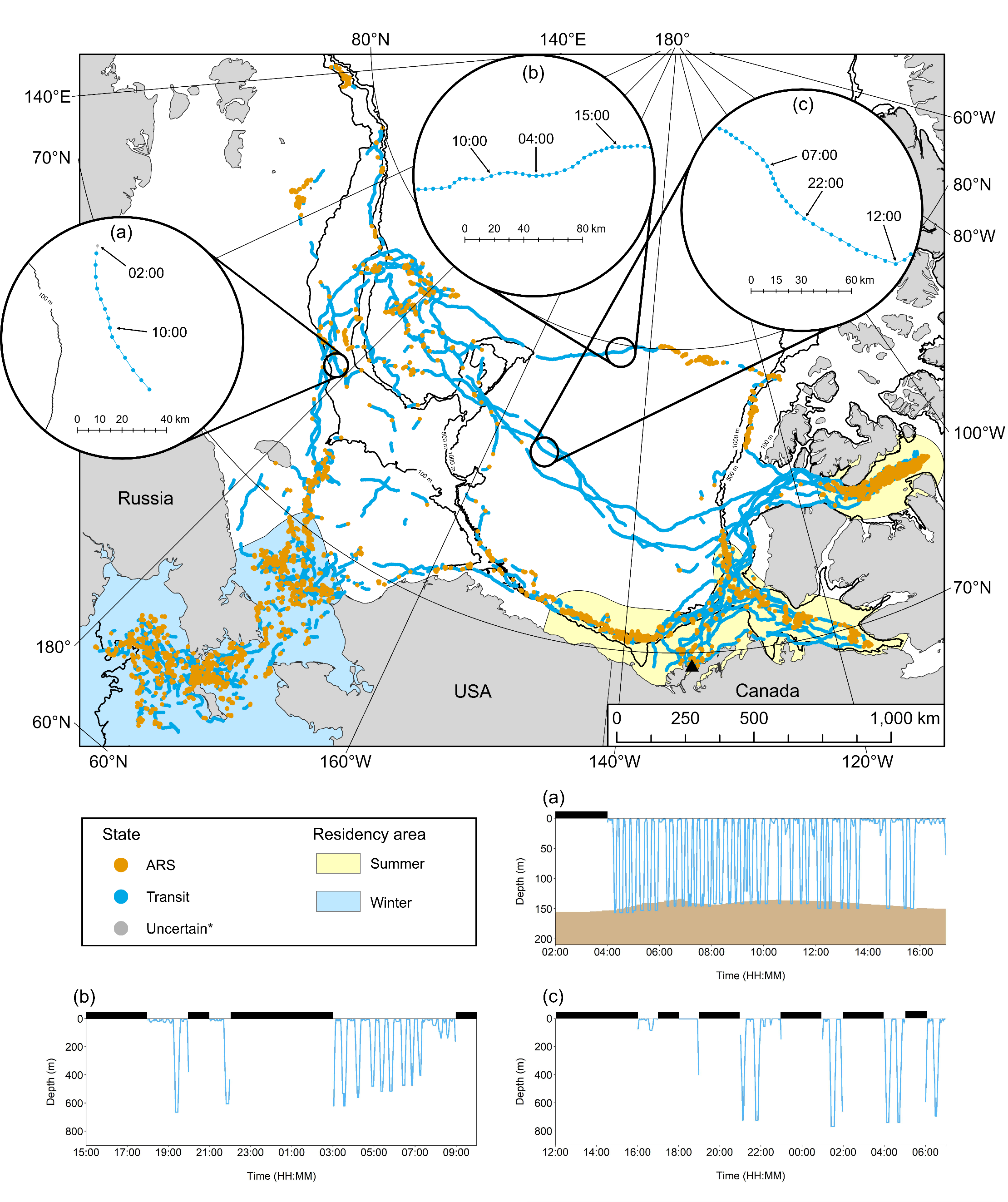


**Figure 5:** State-decoded locations recorded by belugas between July 2018 and June 2019. The long-distance movement phase analysed includes all locations not bounded by the summer or winter residency area polygons. ‘Uncertain*’ locations (probability of < 0.9 of being in ARS or Transit) shown in zoomed inset panels a-c only. Zoomed inset panels a-c show examples of state-decoded beluga tracks, with the corresponding dive profiles colour-coded by state shown in the lower panels. (a) beluga LC2018#1 10^th^ November 2018 between 02:00-17:00 UTC, (b) beluga LC2018#4 15th September 2018 at 15:00 UTC to 16th September 2018 at 10:00 UTC, (c) beluga LC2018#8 25^th^ August 2018 at 12:00 UTC to 26^th^ August 2018 at 07:00 UTC. Black bars above dive profiles denote periods with missing data and the seafloor depth is shaded brown at the bottom. Note, zoomed inset panels only show the individual beluga referred to for that period. Note that the shallowest dives (up to 23 m) were not classified as foraging type dives.

**References**

1. Storrie L, Hussey NE, MacPhee SA, O’Corry-Crowe G, Iacozza J, Barber DG, et al. Year-Round Dive Characteristics of Male Beluga Whales From the Eastern Beaufort Sea Population Indicate Seasonal Shifts in Foraging Strategies. Front Mar Sci. 2022 Jan 3;8:715412.
